# NOD2 attenuates osteoarthritis via reprogramming the activation of synovial macrophages

**DOI:** 10.1186/s13075-023-03230-4

**Published:** 2023-12-20

**Authors:** Changchuan Li, Zhuji Ouyang, Yuhsi Huang, Sipeng Lin, Shixun Li, Jing Xu, Taihe Liu, Jionglin Wu, Peidong Guo, Zhong Chen, Haoyu Wu, Yue Ding

**Affiliations:** Department of Orthopaedic Surgery, Sun Yat-Sen Memorial Hospital, Sun Yat-Sen University, Guangzhou, 510120 China

**Keywords:** Synovium, Macrophage, Polarization, NOD2

## Abstract

**Objective:**

Synovial inflammation, which precedes other pathological changes in osteoarthritis (OA), is primarily initiated by activation and M1 polarization of macrophages. While macrophages play a pivotal role in the inflammatory process of OA, the mechanisms underlying their activation and polarization remain incompletely elucidated. This study aims to investigate the role of NOD2 as a reciprocal modulator of HMGB1/TLR4 signaling in macrophage activation and polarization during OA pathogenesis.

**Design:**

We examined NOD2 expression in the synovium and determined the impact of NOD2 on macrophage activation and polarization by knockdown and overexpression models in vitro. Paracrine effect of macrophages on fibroblast-like synoviocytes (FLS) and chondrocytes was evaluated under conditions of NOD2 overexpression. Additionally, the in vivo effect of NOD2 was assessed using collagenase VII induced OA model in mice.

**Results:**

Expression of NOD2 was elevated in osteoarthritic synovium. In vitro experiments demonstrated that NOD2 serves as a negative regulator of HMGB1/TLR4 signaling pathway. Furthermore, NOD2 overexpression hampered the inflammatory paracrine effect of macrophages on FLS and chondrocytes. In vivo experiments revealed that NOD2 overexpression mitigated OA in mice.

**Conclusions:**

Supported by convincing evidence on the inhibitory role of NOD2 in modulating the activation and M1 polarization of synovial macrophages, this study provided novel insights into the involvement of innate immunity in OA pathogenesis and highlighted NOD2 as a potential target for the prevention and treatment of OA.

**Supplementary Information:**

The online version contains supplementary material available at 10.1186/s13075-023-03230-4.

## Introduction

Osteoarthritis (OA), the most prevalent joint disease, stands as a primary cause of joint pain and disability [1]. Featured by comprehensive joint lesions including cartilage degradation, synovial inflammation, osteophyte formation and subchondral bone sclerosis, OA significantly impacts life quality, labor ability, and life expectancy. Clinically, the knee is the most common site of OA, followed by the hand and hip. The prevalence of symptomatic knee OA exceeds 10%, with a lifetime risk ranging from 14 to 45% [2]. While limited evidence suggests potential structural modification through pharmacological therapies, synchronous symptom benefits remain elusive [3]. Consequently, OA constitutes a substantial and escalating health burden, posing remarkable implications for patients, healthcare systems, and broader socioeconomic costs [4, 5].

In recent decades, the hypothesis has been widely accepted that OA pathogenesis starts from injury and consequent degradation of cartilage. However, emerging evidence highlights synovial inflammation as a pivotal process [6]. Synovitis, as indicated by magnetic resonance imaging (MRI), is associated with OA symptoms [7]. Synovial inflammation arises from the immune response against damage-associated molecular patterns (DAMPs) or alarmins, primarily high-mobility group box-1 (HMGB1) and proteins from the S100 family [8]. The synovium, predominantly composed of macrophages and fibroblast-like synoviocytes (FLS), underscores the crucial role of synovial macrophage in orchestrating inflammatory processes during OA pathogenesis [9–11].

HMGB1, a prominent DAMP in OA pathogenesis, is released into the synovial fluid by senescent or necrotic cells [12]. HMGB1 stimulates synovial macrophages through pattern recognition receptors (PRRs), notably toll-like receptor 4 (TLR4), activating downstream inflammatory signaling pathways, including NF-κB and MAPK. In response to HMGB1, synovial macrophages release inflammatory factors, such as tumor necrosis factor α (TNF-α), initiating synovial inflammation [13]. Nucleotide-binding oligomerization domain containing 2 (NOD2), a member of PRRs alongside TLR4, is expressed in the cytosol of immune cells, including myeloid cells, monocytes, macrophages, and dendritic cells. NOD2 has been reported to be associated with Crohn’s disease and plays important roles in microbe sensing and host response [14]. Recent findings indicate the inhibitory influence of NOD2 on TLR signaling pathways in colorectal tumorigenesis [15]. However, it remains unknown whether NOD2 has an influence on the pathogenesis of OA.

This study provides compelling evidence for the pivotal role of NOD2 in OA pathogenesis, demonstrating its capacity to mitigate osteoarthritis by attenuating HMGB1-induced synovial macrophage activation. Using collagenase-induced osteoarthritis (CIOA) model in mice, where synovial hyperplasia and the effect of macrophages were more pronounced with significant synovial activation [9], our study elucidates the mechanistic role of NOD2 in orchestrating the activation of synovial macrophages, and highlights NOD2 as a potential target for OA prevention and treatment.

## Materials and methods

### Human synovial tissue

Human synovial tissue was obtained from 13 patients in the Department of Orthopaedic Surgery, at Sun Yat-sen Memorial Hospital, Sun Yat-sen University. The patients underwent arthroscopic surgery or joint replacement of the knee, with ten diagnosed with osteoarthritis. Ethical considerations precluded obtaining synovial membrane samples from completely healthy individuals. To serve as control, synovial membrane samples from patients with acute trauma, devoid of chronic inflammation, were utilized, as supported by relevant literature [16, 17]. Written informed consents were obtained before surgery, with approval from the Ethics Committee of Sun Yat-sen Memorial Hospital (Approval No. SYSEC-KY-KS-2021–243). The procedures were in accordance with the ethical standards of the responsible committee on human experimentation (institutional and national) and with the Declaration of Helsinki. Demographic characteristics are listed in Supplementary Table 1.

### Experimental mouse model

Healthy male C57BL/6 J mice (8 weeks old) were obtained from the Animal Laboratory of Sun Yat-sen University. Randomized using a random number table, they were divided into 4 groups, each with 6 mice, 24 mice in total. Sample size determination was based on our previous experiments [18]. Mice were housed in a specific pathogen-free (SPF) animal care facility, with accessible food and water. Osteoarthritis was induced in the CIOA group by intra-articular injection of collagenase VII (1U) into the right knee joint, twice on alternate days [16]. In the CIOA + Mock/ + NOD2 overexpression (oe-NOD2) group, mock lentiviruses or lentiviruses overexpressing NOD2 were injected into the articular cavity 1 week after collagenase injection [19]. Osteoarthritis Research Society International (OARSI) score, osteophyte formation, and expression of specific proteins were observed. All protocols and experiments were approved by the Institutional Animal Care and Use Committee of Sun Yat-sen University (Approval No. 20220223), in accordance with the animal care guidelines and the 3Rs principle (replacement, refinement, and reduction). To be explicit, confounders were not controlled.

### Immunohistochemical (IHC) staining

Synovial tissue was fixed in 4% paraformaldehyde (PFA), transparentized with xylene and embedded in paraffin at 54 °C for sectioning (section thickness: 3 μm). For knee specimens of mice, 10% ethylenediaminetetraacetic acid (EDTA) (pH 7.4) was used for decalcification for 30 days, embedding in paraffin and sectioning.

Sections underwent deparaffinization and rehydration using xylene and ethanol with gradient concentrations. Antigen retrieval was achieved by pepsin, followed by immersion in 3% H_2_O_2_ for 20 min to eliminate endogenous peroxidase activity, and blocking with 5% bovine serum albumin (BSA) for 1 h. Tissue sections were incubated with diluted primary antibodies for 2 h at 37 °C, followed by incubation with diluted secondary antibodies labeled with horseradish peroxidase (HRP) for 30 min. Visualization with 3,3′-diaminobenzidine (DAB) and nuclear staining with hematoxylin were performed, and sections were sealed with gum and observed using a biomicroscope (DM2000, Leica).

### Immunofluorescent staining

Tissue sections underwent the same processing as described in the “Immunohistochemical (IHC) staining” section. For macrophages, cells were seeded in confocal plates (BDD011035, Jet Bio-Filtration) and stimulated with HMGB1 at various time points after 24 h. The cells were fixed in 4% PFA for 15 min, incubated in 0.1% Triton X-100 at 20 °C for 15 min, and then gently shaken before blocking with 1% BSA. Following these steps, the sections or cells were subjected to overnight incubation with primary antibodies at 4 °C, and subsequent incubation with secondary antibodies at 20 °C for 1 h. Nuclear staining was achieved using 4′,6-diamidino-2-phenylindole (DAPI), and fluorescence was observed using a confocal microscope (LSM 710, Carl Zeiss).

### Cell preparation of macrophages and fibroblasts

Femurs and tibias from 8-week-old male C57BL/6 J mice were isolated for bone marrow-derived macrophages (BMDMs) extraction in sterile environment. BMDMs were cultured with macrophage colony-stimulating factor (M-CSF) (51,112-MNAH, Sino Biological Inc) for 7 days before further experiments.

BMDMs were cultured in high-glucose Dulbecco’s modified Eagle’s medium (DMEM) with 10% fetal bovine serum (FBS), supplemented with 2% penicillin and streptomycin, at 37 °C in 5% CO_2_. NIH3T3 fibroblasts (CL-0171, Procell) were cultured similarly. At a confluence of approximately 70–80%, cells were stimulated with recombinant human HMGB1 and/or muramyl dipeptide (MDP) at a concentration of 1 μg/ml. Proteasomal degradation was blocked by MG132 (12.5 μM) incubation for 2 h before stimulants were added.

To assess the influence of macrophages on fibroblasts and chondrocytes, macrophages were stimulated with HMGB1 (1 μg/ml) for 24 h as well as macrophages transfected with mock or oe-NOD2 lentiviruses. After centrifugation to remove cell debris, supernatants were applied to fibroblasts or chondrocytes, with the supernatants of unstimulated macrophages as negative control.

### Isolation and culture of chondrocytes

Articular surfaces of femur and tibia were isolated from 4-week-old male Sprague–Dawley rats from the Animal Laboratory of Sun Yat-sen University. Chondrocytes were isolated via incubation with 0.25% trypsin at 37 °C for 20 min and collagenase II at 37 °C for 30 min. The isolated chondrocytes from filtration and centrifugation were resuspended in DMEM/F-12 supplemented with 10% FBS and 2% penicillin and streptomycin.

### Real-time PCR

Total RNA was obtained using the RNAiso Plus reagent kit, and its concentration was determined by NanoDrop™ 2000 spectrophotometer (Thermo Fisher Scientific). RNA solution was mixed with the PrimeScript™ RT Master Mix reagent kit for reverse transcription, and the cDNA solution was further mixed with UNICON™ qPCR SYBR® Green Master Mix and corresponding primers for real-time polymerase chain reaction (PCR) on LightCycler® 96 Real-Time PCR System (Roche). 2^−ΔΔCt^ formula was applied for the quantitative analysis of gene expression. Primer sequences for PCR and information on reagents in this study were demonstrated in Supplementary Table 2 and Supplementary Table 3, respectively.

### Western blotting

Cells were treated with lysate composed of radioimmunoprecipitation assay (RIPA) buffer, phenylmethylsulphonyl fluoride (PMSF), and phosphatase/protease inhibitor cocktail. Protein concentration was measured using a bicinchoninic acid (BCA) assay kit, and approximately 20 μg of protein was applied to sodium dodecyl-sulfate polyacrylamide gel electrophoresis (SDS-PAGE) and transferred to a polyvinylidene difluoride (PVDF) membrane. The membrane was sequentially immersed in 5% BSA at room temperature for 1 h, primary antibody at 4 °C overnight, and then HRP-conjugated secondary antibody at room temperature for 1.5 h. Super ECL Detection Reagent was applied to the membrane, and immunoblotting data were obtained via the digital imaging system (G:BOX Chemi XT4, Syngene). Semi-quantitative analysis of western blotting was performed using ImageJ software (Release 1.53p).

#### ELISA

Supernatants from the cell culture system were collected, and cell debris was removed via centrifugation. TNF-α levels were measured with an enzyme-linked immunosorbent assay (ELISA) kit, according to the producer’s instructions.

### siRNA and lentiviruses

To genetically modify the expression of NOD2 in macrophages, small interfering RNA (siRNA) sequence targeting mouse NOD2 (Gene ID: 257,632) (NOD2-siRNA) was designed and synthesized by GenePharma. Negative control siRNA (NC-siRNA) served as control. Macrophages were transfected with NOD2-siRNA with the assistance of Lipofectamine™ RNAiMAX, after starvation in serum-free OPTI-MEM™ for 1 h. Six hours after transfection, the medium was replaced by DMEM with 10% FBS.

Recombinant lentivirus targeting mouse NOD2 (NOD2-LV) was constructed by Cyagen, with mock as the control. Macrophages at a confluence of approximately 30% were incubated with lentiviruses at 37 °C overnight, and subjected to fluorescent cell sorting, to obtain stable transfected macrophages. Similar methods were used for targeted inhibition or overexpression of NLRP12 in macrophages. Sequences of siRNAs were demonstrated in Supplementary Table 4.

### Flow cytometry

Macrophages were stimulated, harvested, and isolated by centrifugation, followed by incubation with eBioscience™ IC Fixation Buffer at 20 °C for 20 min, and later with Perm/Wash Buffer to increase permeability. Macrophages were then incubated with APC-conjugated CD206 antibody, or PE-Cyanine7-conjugated iNOS antibody, for 20 min at 20 °C. Further analysis of macrophages was performed with the assistance of flow cytometry instruments (FACSVerse™, BD Biosciences).

### RNA-seq

Macrophages were stimulated with HMGB1 for 4 h and lysed using the TRIzol™ Reagent to obtain total RNA. After removing rRNAs, mRNAs and ncRNAs were retained for strand-specific construction and sequencing. RNA fragments were reversely transcribed into cDNAs, and PCR was applied for amplification. Illumina HiSeq 4000 was employed for sequencing, and raw reads containing the adapter or demonstrating low quality (*Q*-value ≤ 20) were removed. The remaining reads were mapped to the reference genome of mouse (GRCm38). Differentially expressed genes (DEGs) were identified when FDR < 0.05 and |log_2_ FC|> 1.

### Micro-CT

Whole knee joints were fixed in 4% PFA and assessed using micro-computed tomography (micro-CT) with an imaging system (ZKKS-MCT-Sharp, Zhongke Kaisheng Medical Technology). Radiographic parameters were set at 70 kV and 100 μA within 100 ms (section thickness 10 μm) to obtain optimal projections. The region of interest (ROI) was designated from the images and processed with ZZKS-MicroCT4.1 software, to obtain qualitative data on the quantity and volume of osteophytes around the knee joints.

### Histological assessment

Safranin O/fast green staining was conducted, and a scoring system developed by OARSI was adopted for semi-qualitative analysis of knee joints, considering the extent and depth of cartilage loss to assess the extent and severity of cartilage lesions. These assessments were performed by PDG and THL, who were blinded to group assignment.

Quantification of IHC staining was performed using the ImageJ software (National Institutes of Health, Bethesda, Maryland), with IHC Profiler plugin of ImageJ. Randomly selected fields of IHC images were divided into four groups based on pixel intensity values: high positive, positive, low positive, and negative. The following formula was used to calculate the IHC optical density score (ODS) for the IHC images. ODS = (high positive [%] × 4 + positive [%] × 3 + low positive [%] × 2 + negative [%] × 1)/100, where % represents the percentage contribution.

### Migration and invasion

To evaluate the influence of macrophages on fibroblasts, wound healing, migration and invasion of NIH3T3 fibroblasts were assessed by scratch assay and transwell assay, respectively. Macrophages were stimulated for 24 h with 1 μg/ml HMGB1 as well as macrophages transfected with mock or oe-NOD2 lentiviruses. Supernatants were collected and centrifuged to discard cell debris. Supernatants of unstimulated macrophages were collected as control. The NIH3T3 monolayer was scratched by a 200-μl pipette tip in a uniform pattern, washed with PBS to remove detached cells, and then incubated in the supernatants for 24 h. Images were acquired for quantitative analysis using the ImageJ software (Release 1.53p), and the area where no cells were attached was calculated. The migration rate was expressed as (1 − final area/initial area) × 100%.

Transwell® with 8.0 μm pores (3422, Corning Incorporated) coated with Matrigel® were used to assess invasion of NIH3T3 fibroblasts. Cells were seeded in the upper chambers with serum-free medium, and supernatants were added to the lower chambers. After 48 h of incubation, cells in the upper chambers were wiped off with a cotton swab. The cells on the opposite side of the upper chambers were fixed with 4% PFA and stained with solution of crystal violet. The quantity of stained cells was calculated as the average number among three randomly chosen areas.

### Statistics

The normality of data was tested using the Shapiro–Wilk test. For non-parametric analyses, the Wilcoxon test was performed for comparison between two groups, and the Kruskal–Wallis test followed by Dunn’s multiple comparisons test was used for multi-group comparisons. Sample medians and interquartile ranges were presented. For parametric analyses, Student’s *t* test was performed for comparison between two groups, and analysis of variance (ANOVA) with Bonferroni’s correction was used for multi-group comparison. Data were presented as means and standard errors of the mean (s.e.m.). All analyses were performed using the GraphPad Prism 6 software (GraphPad Software). *P* < 0.05 was considered as significant.

## Results

### NOD2 is upregulated in synovial macrophages of osteoarthritis patients and HMGB1-stimulated macrophages

**Fig. 1:**
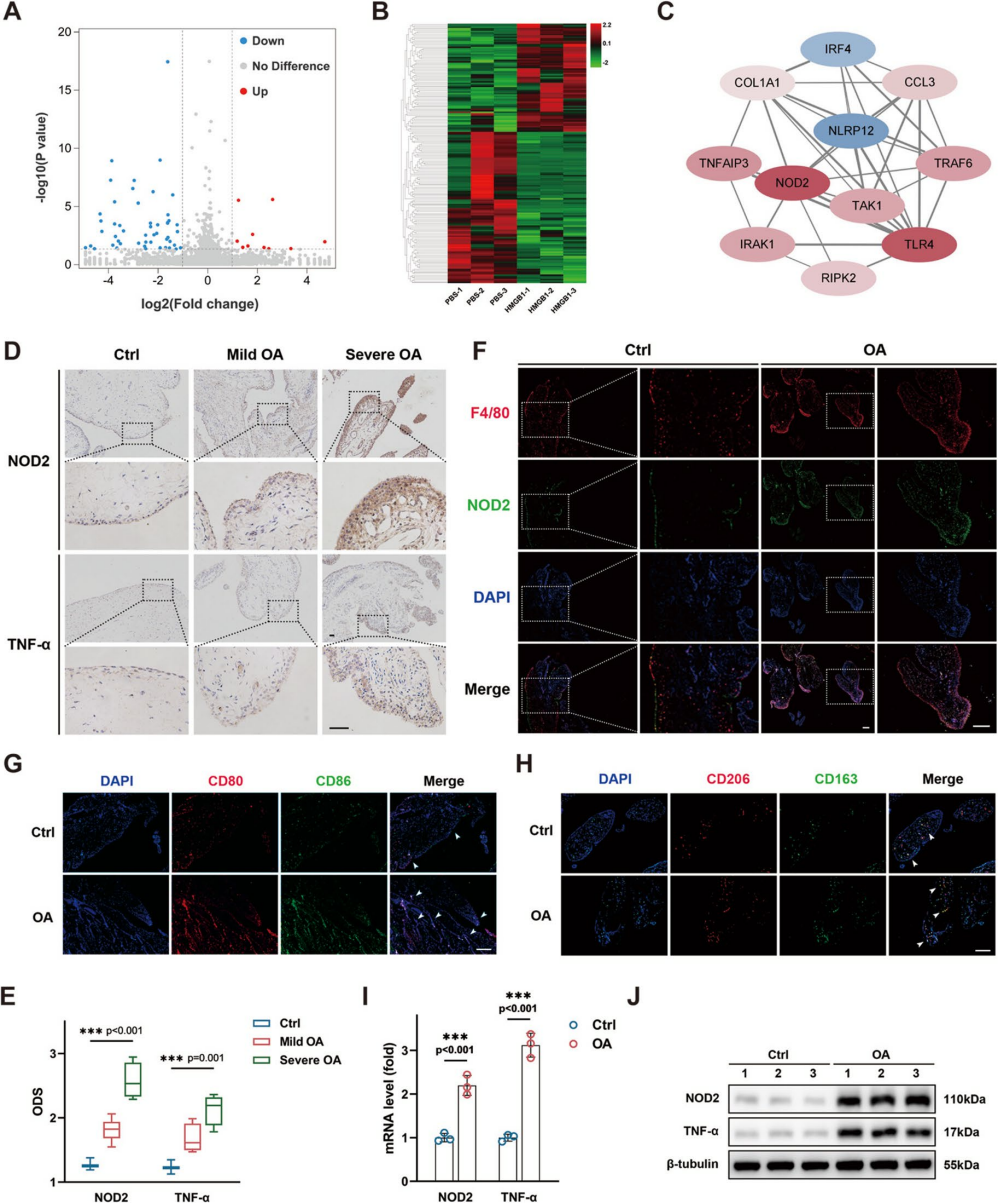
NOD2 is upregulated in synovial macrophages of osteoarthritis patients. **A** Volcano plot demonstrating differentially expressed genes in macrophages stimulated by HMGB1 for 4 h. **B** Heat map and cluster analysis of RNA-seq data. **C** Protein–protein interaction network indicating the association of NOD2 with HMGB1/TLR4 signaling. **D** Expression of NOD2 and TNF-α in osteoarthritic synovial tissue compared with non-osteoarthritic tissue (scale bar = 100 μm). **E** Intensity score of NOD2 and TNF-α in osteoarthritic synovial tissue compared with non-osteoarthritic synovial tissue. **F** Co-localization of NOD2 with F4/80, a macrophage marker (scale bar = 200 μm). **G**, **H** M1/M2 ratio favoring M1 subtype transformation in osteoarthritis synovial tissue. **I** Elevated mRNA expression of NOD2 and TNF-α in osteoarthritic synovial tissue. **J** Elevated expression of NOD2 and TNF-α at protein level in osteoarthritic synovial tissue. *p < 0.05, **p < 0.01, and ***p < 0.001. Data were presented as boxplots (center line, median; box limits, 25 to 75th percentiles; whiskers, min to max), or mean ± s.e.m. values. *n* = 3 biologically independent replicates for PCR. Kruskal–Wallis test followed by Dunn’s multiple comparisons test for ODS scores of IHC images and Student’s *t* test for PCR

To investigate potential molecules orchestrating macrophage activation, we subjected RAW264.7 macrophages to HMGB1 stimulation for 4 h, to capture mRNA profiles through RNA sequencing. DEGs were identified (|log_2_ FC|> 1, FDR < 0.05), and the results were visually represented through heat map and cluster analysis (Fig. 1A, B). Protein–protein interaction network (PPIN) based on the STRING database unveiled a close association between NOD2 and TLR4 pathway activation in macrophages (Fig. 1C).

We further collected synovial tissue samples from osteoarthritis patients and healthy controls, for IHC staining, with demographic characteristics outlined in Supplementary Table 1. In accordance with the RNA-seq, the expression of NOD2 exhibited a substantial increase in osteoarthritic synovial tissue, compared to healthy synovial tissue. Concurrently, the well-known proinflammatory cytokine TNF-α, primarily secreted by activated macrophages, demonstrated an upregulation in osteoarthritic synovial tissue (Fig. 1D). Quantitative Bresalier’s analysis confirmed elevation of NOD2 and TNF-α level, by comparing the average intensity scores between the two groups (Fig. 1E). To explore the spatial distribution of NOD2 within synovial tissue, immunofluorescent staining was employed. F4/80, a surface marker of macrophages, exhibited elevated expression in osteoarthritic synovial tissue, mirroring the pattern of NOD2. Remarkably, the green fluorescence indicative of NOD2 showed consistent co-localization with the red fluorescence of F4/80, signifying macrophages as the principal residence of NOD2 in osteoarthritic synovial tissue (Fig. 1F). Immunofluorescent staining further indicated a shift in the M1/M2 ratio favoring M1 subtype transformation in osteoarthritic synovial tissue, compared with healthy controls (Fig. 1G, H). Validation through real-time PCR and Western blotting of synovial tissue corroborated elevated expression of NOD2 and TNF-α in osteoarthritis patients (Fig. 1I, J, Supplementary Fig. 1A, B). Primer sequences for PCR and information on reagents utilized in this study are detailed in Supplementary Table 2 and Supplementary Table 3, respectively.

### HMGB1 promotes NOD2 expression and macrophage activation

**Fig. 2:**
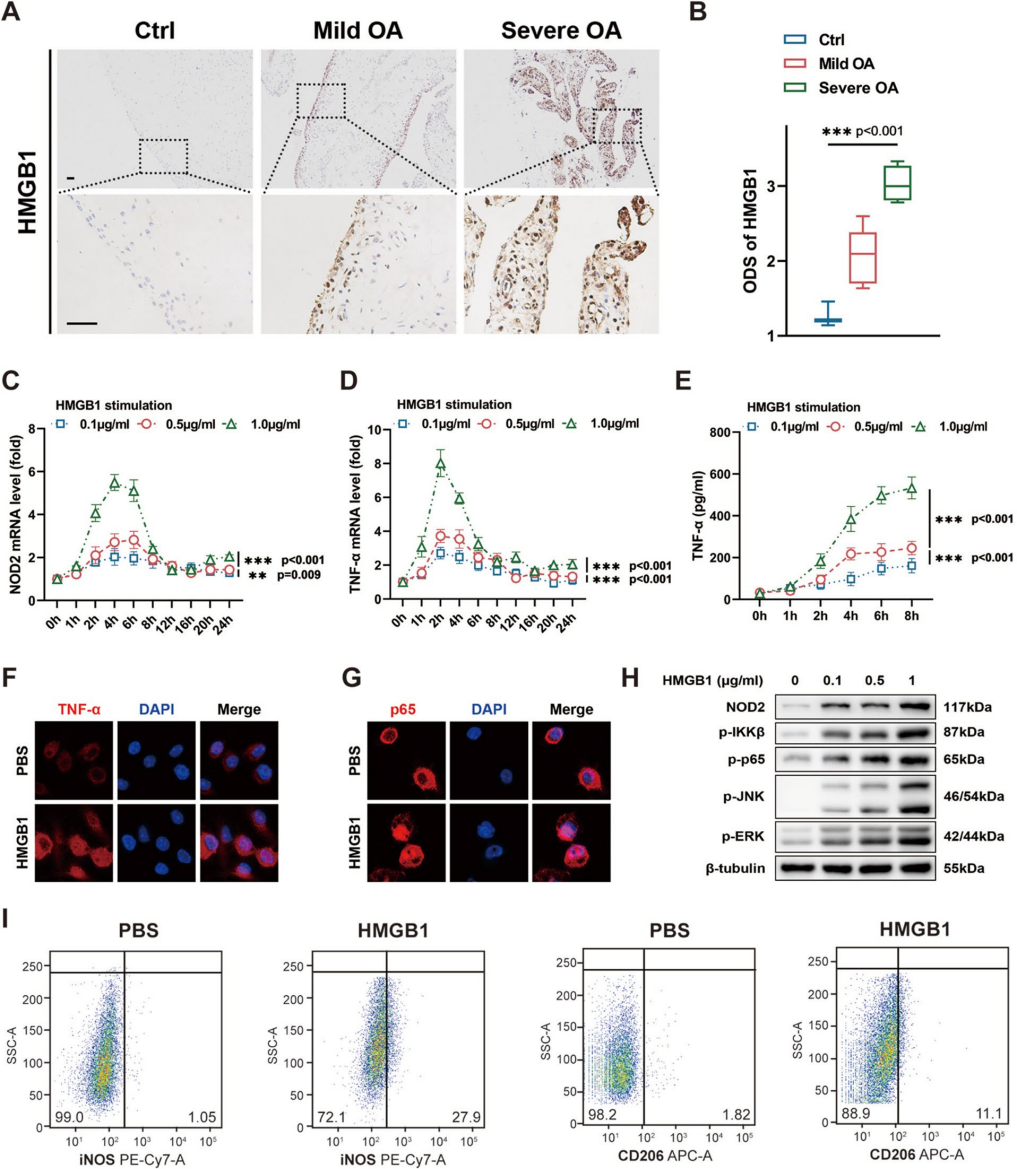
HMGB1 promoted NOD2 expression and macrophage activation. **A**, **B** Intensity score of HMGB1 in osteoarthritic and non-osteoarthritic synovial tissue (scale bar = 100 μm) (*n* = 3 in Ctrl group, *n* = 6 in mild OA group, and *n* = 4 in severe OA group). **C** NOD2 expression at mRNA level in response to HMGB1. **D** TNF-α expression at mRNA level in response to HMGB1. **E** Enhanced secretion of TNF-α from macrophages in response to HMGB1. **F** Increased TNF-α expression at protein level in macrophages stimulated by HMGB1. **G** HMGB1-induced translocation of p65 from cytoplasm to nucleus. **H** Activation of NF-κB and MAPK pathway in macrophages upon HMGB1 stimulation. **I** M1 polarization of macrophages induced by HMGB1. *p < 0.05, **p < 0.01, and ***p < 0.001. Data were presented as boxplots (center line, median; box limits, 25 to 75th percentiles; whiskers, min to max), or mean ± s.e.m. values. *n* = 3 biologically independent replicates for in vitro experiments. Kruskal–Wallis test followed by Dunn’s multiple comparisons test for ODS scores of IHC images and two-way ANOVA with Bonferroni’s test for PCR

IHC staining of synovial tissue unveiled an elevated expression of HMGB1 in osteoarthritis patients, particularly in cases classified as radiographically severe osteoarthritis (Kellgren/Lawrence classification > 2) (Fig. 2A). This observation was substantiated by comparing the average intensity scores across different groups (Fig. 2B). Subsequently, we investigated the impact of HMGB1 stimulation on the expression of NOD2 and TNF-α, an inflammatory cytokine, in macrophages at various time intervals. HMGB1 stimulation at gradient concentrations ranging from 0.1 μg/ml to 1.0 μg/ml significantly upregulated NOD2 mRNA expression, with a more pronounced effected noted at higher concentrations of HMGB1 (Fig. 2C). Consistently, real-time PCR depicted an increase in TNF-α expression at mRNA level following HMGB1 stimulation (Fig. 2D). This elevation was further validated at protein level by enzyme-linked immunosorbent assay (ELISA) assay and immunofluorescent staining (Fig. 2E, F). Immunofluorescent staining additionally illustrated the translocation of p65 (red) from the cytoplasm into the nucleus (blue) in macrophages upon HMGB1 stimulation (Fig. 2G).

Indeed, HMGB1 stimulation not only led to a significant increase in the protein expression of NOD2 but also induced elevated levels of p-IKKβ, p-p65, p-JNK, and p-ERK, indicative of a comprehensive activation of the NF-κB and MAPK pathways (Fig. 2H, Supplementary Fig. 1C-G). Activated macrophages typically undergo polarization into either M1 or M2 subtype. By employing biomarkers such as iNOS (characteristic of M1 subtype) and CD206 (characteristic of M2 subtype), flow cytometry indicated that HMGB1 induced M1 polarization of macrophages (Fig. 2I).

### NOD2 modulates macrophage activation induced by HMGB1

**Fig. 3:**
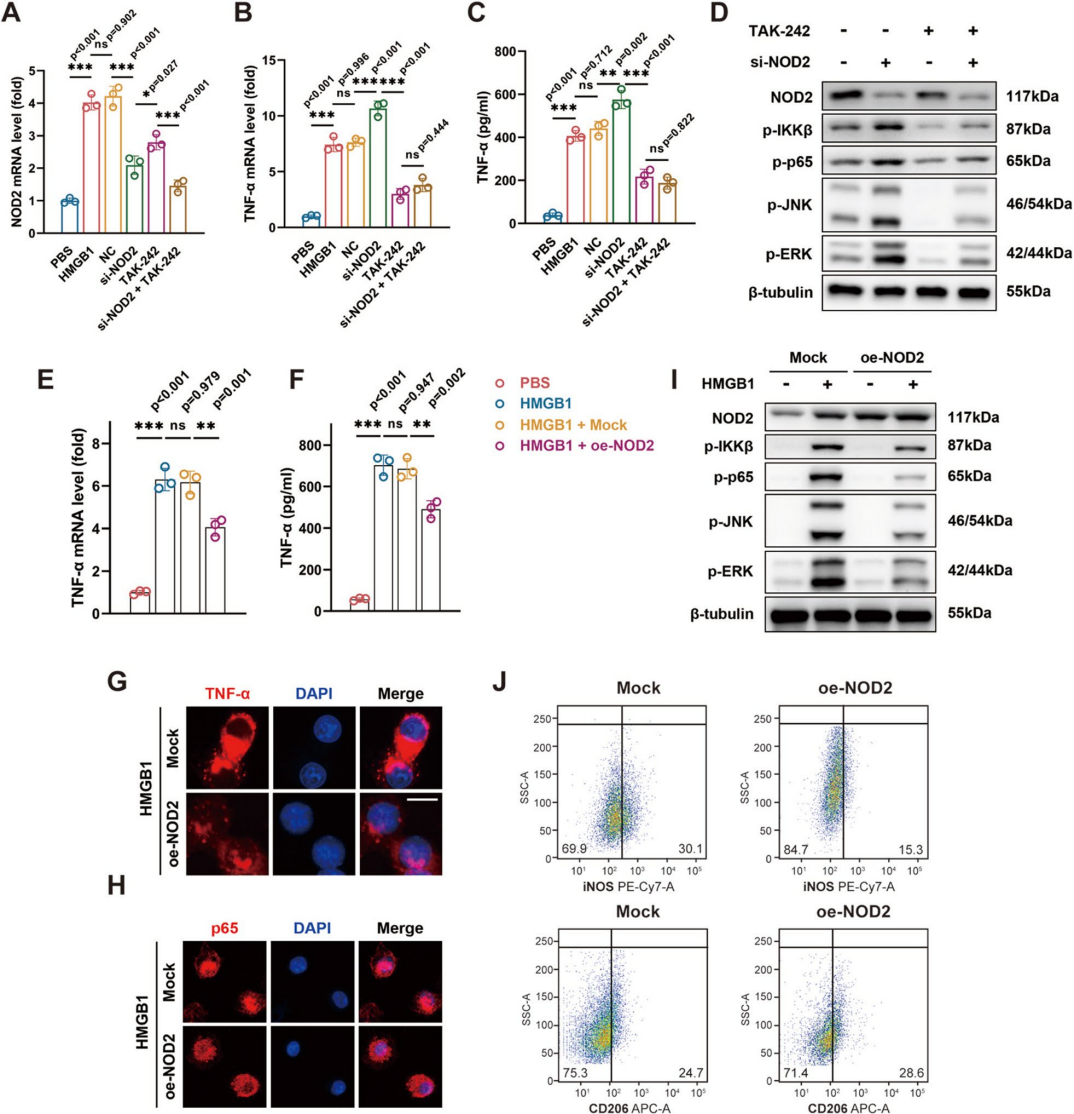
NOD2 exerts inhibitory effect on HMGB1 induced macrophage activation. **A** Decreased mRNA level of NOD2 by siRNA targeting NOD2 (si-NOD2) and TAK-242. **B**, **C** Suppressed mRNA and protein level of TNF-αby TAK-242, while increased by si-NOD2. **D** Inhibition of NF-κB and MAPK pathway by TAK-242 but activation by si-NOD2. **E**, **F** Lower TNF-α expression by lentivirus-mediated NOD2 overexpression (oe-NOD2). **G**, **H** Weakened TNF-α production and p65 translocation in the oe-NOD2 group. **I** Dampened activation of NF-κB and MAPK pathway in macrophages overexpressing NOD2. **J** Impaired M1 polarization of macrophages in the oe-NOD2 group. *p < 0.05, **p < 0.01, and ***p < 0.001. Data were presented as mean ± s.e.m. values. *n* = 3 biologically independent replicates for in vitro experiments. Student’s *t* test or one-way ANOVA with Bonferroni’s test for PCR

To elucidate the influence of NOD2 on macrophage activation induced by HMGB1, we employed siRNA targeting NOD2 (si-NOD2) in macrophages. Detailed designation, synthesis, and construction of si-NOD2 have been described in our previous study [20], and the sequences are provided in Supplementary Table 4, with verification data presented in Supplementary Fig. 4. Taking it into consideration that TLR4 is the predominant receptor for HMGB1, we applied TAK-242, an inhibitor of the TLR4 pathway, to explore potential interaction between NOD2 and TLR4. Real-time PCR confirmed significant reduction in NOD2 expression in macrophages transfected with si-NOD2. Intriguingly, TLR4 pathway inhibition also led to a decrease in NOD2 mRNA expression, highlighting the pivotal role of TLR4 in macrophage NOD2 upregulation in response to HMGB1 (Fig. 3A). As expected, TLR4 inhibition resulted in a reduction in TNF-α expression at both mRNA and protein levels. Notably, TNF-α expression in NOD2 knock-down macrophages was significantly higher than the negative control (NC) group, suggesting an inhibitory role of NOD2 in HMGB1-induced macrophage activation (Fig. 3B, C). This was further confirmed by Western blotting, where TAK-242 attenuated NOD2 regulation, and NOD2 knock-down resulted in a more significant activation of the NF-κB and MAPK pathways (Fig. 3D, Supplementary Fig. 2A-E).

Next, we further explore the regulatory effect of NOD2 on macrophage activation, by constructing recombinant lentivirus carrying sequences that induced NOD2 overexpression, as detailed in our previous study [20]. Macrophages overexpressing NOD2 demonstrated lower TNF-α expression, both at mRNA and protein levels, in response to HMGB1 stimulation (Fig. 3E, F). This was corroborated by immunofluorescent staining (Fig. 3G). Notably, MDP, a potent activator of NOD2, failed to inhibit TNF-α expression (Supplementary Fig. 2F, G). Furthermore, NOD2 overexpression in macrophages impeded the translocation of p65 (red) from the cytoplasm into the nucleus (blue) induced by HMGB1 (Fig. 3H). Moreover, NOD2 overexpression reduced the protein levels of p-IKKβ, p-p65, p-JNK, and p-ERK in response to HMGB1 (Fig. 3I, Supplementary Fig. 2H-L) and dampened M1 polarization of macrophages (Fig. 3J).

### NOD2 overexpression attenuates the pro-inflammatory paracrine effect of macrophages on FLS and chondrocytes

**Fig. 4:**
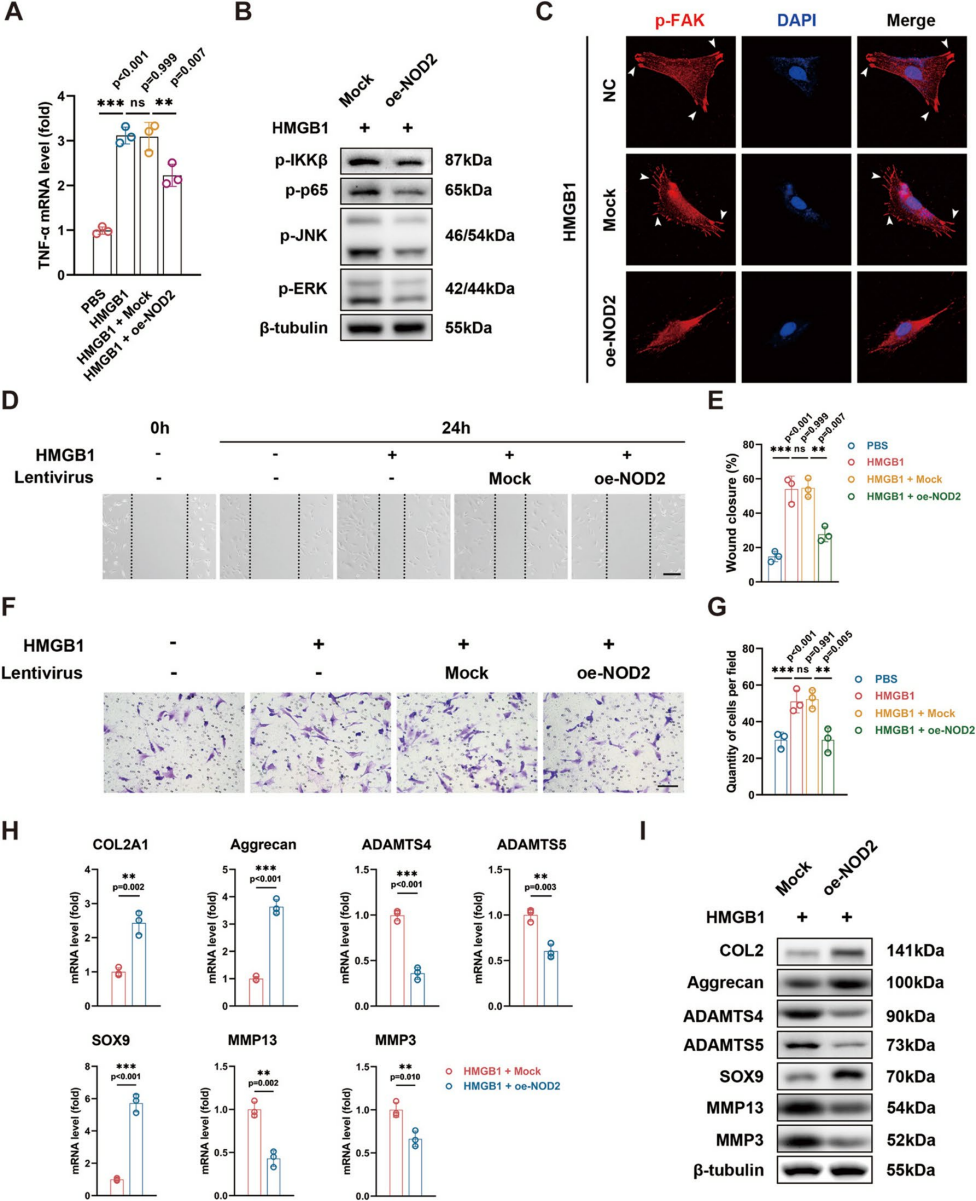
NOD2 overexpression hampers the pro-inflammatory paracrine effect of macrophages on FLS and chondrocytes. **A**, **B** Dampened activation of NF-κB and MAPK pathway and TNF-α production in FLS by oe-NOD2. **C** FLS develops polarized formation of lamellipodia stained positive for p-FAK, while lacking in the oe-NOD2 group. **D**, **E** Reduced migration of FLS in the oe-NOD2 group. **F**, **G** Attenuated invasion of FLS in the oe-NOD2 group. **H**, **I** Elevated anabolic factors and decreased catabolic factors in the oe-NOD2 group. *p < 0.05, **p < 0.01, and ***p < 0.001. Data were presented as mean ± s.e.m. values. *n* = 3 biologically independent replicates for in vitro experiments. Student’s *t* test or one-way ANOVA with Bonferroni’s test for PCR, wound closure, as well as transwell assays

To unravel the paracrine effects of NOD2-overexpressing macrophages on FLS and chondrocytes, macrophages were exposed to 1 μg/ml HMGB1 for 24 h, as well as macrophages transfected with mock or oe-NOD2 lentiviruses, with unstimulated macrophages as negative control. Supernatants were collected, centrifuged to eliminate cell debris, and subsequently applied to fibroblasts (Fig. 4A–G) and chondrocytes (Fig. 4H–I). Compared to the untransfected and mock groups, real-time PCR demonstrated lower mRNA level of TNF-α in fibroblasts of the oe-NOD2 group (Fig. 4A). This aligns with the results from Western blotting, where subdued activation of the NF-κB and MAPK pathways was observed in the oe-NOD2 group (Fig. 4B, Supplementary Fig. 3A). Notably, fibroblasts developed polarized formation of lamellipodia, which was marked by p-FAK, and indicative of a proinflammatory phenotype with invading capability. Conversely, these features were absent in the oe-NOD2 group (Fig. 4C). Migration and invasion of fibroblasts, assessed by scratch assay and transwell assay, were also impaired in the oe-NOD2 group (Fig. 4D–G).

Moreover, the overexpression of NOD2 in macrophages significantly enhanced the expression of anabolic factors in chondrocyte metabolism, including COL2A1 (or COL2 protein), SOX9, and aggrecan. Simultaneously, the expression of catabolic factors in chondrocyte metabolism, such as MMP3, MMP13, ADAMTS4, and ADAMTS5, was downregulated (Fig. 4H–I, Supplementary Fig. 3B).

### NOD2 alleviates pathological changes in mouse osteoarthritis model

**Fig. 5:**
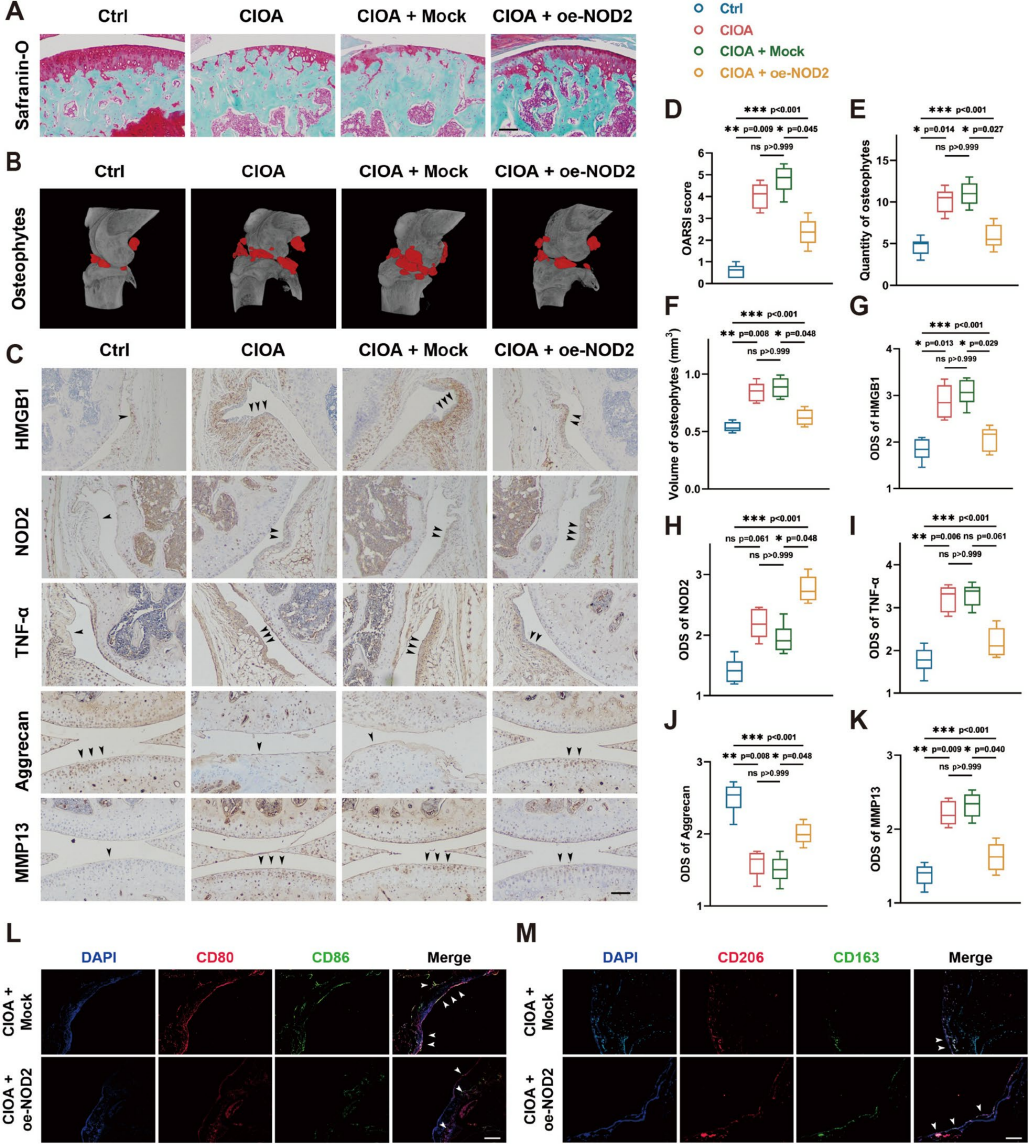
NOD2 alleviates pathological changes in mouse osteoarthritis model. **A** Safranin O/fast green staining of knee joints after intra-articular injection (scale bar = 100 μm). **B** Micro-CT scanning and 3D reconstruction of knee joints after intra-articular injection. **C** IHC staining of knee joints after intra-articular injection (scale bar = 100 μm). **D** OARSI score indicating cartilage injury and its mitigation by oe-NOD2 lentiviral injection. **E**, **F** Quantitative analysis of micro-CT shows osteophyte formation and attenuation by oe-NOD2. **G**, **K** Intensity score analysis of IHC staining demonstrating the impact of oe-NOD2 on synovial tissue and articular cartilage. **K**–**M** Association of interrupted M1 phenotype transition with the alleviation of osteoarthritis by NOD2 overexpression. *p < 0.05, **p < 0.01, and ***p < 0.001. Data were presented as boxplots (center line, median; box limits, 25 to 75th percentiles; whiskers, min to max) (*n* = 6 for each group). Kruskal–Wallis test followed by Dunn’s multiple comparisons test for multi-group comparisons of OARSI scores, osteophytes and ODS scores of IHC images

To assess the impact of NOD2 in osteoarthritis, we constructed CIOA mouse model, with detailed grouping outlined in the “Materials and methods” section. Data from 6/6 mice were included in each analysis. Intra-articular injection of collagenase VII induced cartilage lesions in the knee joint of mouse, as depicted by Safranin O/fast green staining (Fig. 5A). Lentiviruses yielding overexpression effect of NOD2 significantly preserved articular cartilage, as evidenced by OARSI score (Fig. 5D). Micro-CT scanning and 3D reconstruction unveiled higher quantity and volume of peri-articular osteophytes in the CIOA (0.848mm^3^, 95% CI 0.760–0.935 mm^3^) and CIOA + Mock group (0.885mm^3^, 95% CI 0.795–0.975 mm^3^), compared with the Ctrl group (0.539mm^3^, 95% CI 0.494–0.583 mm^3^). However, intra-articular injection of lentiviruses overexpressing NOD2 alleviated osteophyte formation in the CIOA + oe-NOD2 group (0.622mm^3^, 95% CI 0.547–0.698 mm^3^) (Fig. 5B, E, F).

IHC staining revealed higher expression of HMGB1 in mouse synovial tissue of the CIOA group, compared to the Ctrl group, consistent with the findings in human. Notably, the CIOA + oe-NOD2 group showed impaired elevation of HMGB1, suggesting a negative feedback mechanism on HMGB1 release by NOD2 overexpression (Fig. 5C, G). Further insights into the effect of NOD2 on the pathological process of CIOA in mice were obtained from IHC staining of knee joints. Expression of NOD2 in the synovial tissue was upregulated in the CIOA group and expectedly higher in the CIOA + oe-NOD2 group (Fig. 5C, H). Concurrently, the elevation of TNF-α observed in CIOA was partially dampened by NOD2 overexpression (Fig. 5C, I), indicating an inhibitory effect of NOD2 on the inflammatory process. Moreover, the predominant proteoglycan in articular cartilage, aggrecan, was partially protected by NOD2 overexpression from significant loss in CIOA (Fig. 5C, J). Conversely, NOD2 overexpression exerted an inhibitory effect on MMP-13, a vital catabolic factor involved in cartilage degradation (Fig. 5C, K).

Further exploration of the underlying mechanism by which overexpression of NOD2 alleviated osteoarthritis in mice involved immunofluorescent staining. Both F4/80 and iNOS in synovial tissue were elevated in CIOA, with a more significant increase observed in iNOS, indicating that M1 macrophages were the dominant phenotype in OA. Interestingly, NOD2 overexpression more significantly retarded the increase in iNOS compared to F4/80, suggesting an association between interrupted M1 phenotype transition and the alleviation of osteoarthritis by NOD2 overexpression (Fig. 5L, M).

## Discussion

This study focused on the pivotal role of NOD2 in the pathogenesis of OA. NOD2 as a differentially expressed gene in activated macrophages, as suggested by RNA sequencing, was further corroborated by IHC staining of synovial tissue sections. Elevated in response to HMGB1 stimulation, NOD2 demonstrates a negative impact on macrophage activation and the release of inflammatory cytokines. Subsequent coculture with supernatants from genetically modified macrophages induces phenotypic shift in FLSs and chondrocytes, implicating NOD2 as a pivotal factor that empowers synovial macrophages to orchestrate the inflammatory processes during OA pathogenesis. Furthermore, in vivo overexpression of NOD2 via lentivirus injection significantly alleviates the severity of osteoarthritis in mice. These findings shed light on a novel regulatory element in OA pathogenesis and suggest NOD2 as a potential preventive and therapeutic target.

Recent clinical studies have identified synovial inflammation as a characteristic feature in OA development and progression [7, 21]. Synovial inflammation is detected throughout the entire process of OA development [22], preceding other pathological changes of OA, as a predictive marker before the onset of OA. Macrophages are profoundly involved in various diseases including developmental, inflammatory, tumoral, and degenerative diseases [23, 24]. Direct in vivo evidence of macrophage involvement in human osteoarthritis has been validated, by revealing the fact that activated, not resting macrophages, were recruited in 76% of OA knees [24]. Therefore, understanding the regulatory mechanism of macrophage activation is crucial in OA pathogenesis. In this study, murine BMDMs were employed, utilizing RNA sequencing to identify differentially expressed genes in activated macrophages compared to resting macrophages. RMA sequencing, coupled with protein–protein interaction analysis, ultimately led to the focused on NOD2.

NOD2, also known as caspase recruitment domain-containing protein 15 (CARD15), is a member of the NOD-like receptor (NLR) family and thus named NLR with a CARD2 (NLRC2). NOD2 comprises C-terminal leucine-rich repeats (LRR), intermediate nucleotide-binding-domain (NACHT), and N-terminal CARD [25]. Polymorphisms of NOD2 have been associated with Crohn’s disease, an inflammatory bowel disease, and Blau syndrome, an autoinflammatory condition. Further research has revealed that NOD2 is essential for bacterial sensing by specifically recognizing MDP from bacteria, subsequently activating immune response, including inflammatory signaling pathways such as NF-κB and MAPK [26]. Consistent with prior studies, NOD2 is upregulated in response to MDP stimulation and contributes to macrophage activation and the release of inflammatory cytokines [27]. Notably, this study demonstrates that NOD2 expression also rises in response to HMGB1, one of the most relevant DAMPs in OA pathogenesis. Besides, preconditioning with TLR4 inhibitor impairs the upregulation of NOD2, implicating the indispensable role of the TLR4 signal in HMGB1-induced NOD2 upregulation. However, the specific mechanism underlying NOD2 upregulation via the TLR4 signal remains to be elucidated.

Intriguingly, in lentivirus-transfected macrophages overexpressing NOD2, we observed an unexpected attenuation of the inflammatory response against HMGB1, while the activator of NOD2, MDP, failed to exert an inhibitory effect on the inflammatory response. These seemingly contradictory findings point to the dual effects of NOD2 on HMGB1/TLR4 signaling and the subsequent inflammatory response, a mechanism that remains mechanistically unclear. Existing literature suggests that NOD2 suppresses TLR-mediated activation of NF-κB signaling pathway via interferon regulatory factor 4 (IRF4), which can be induced in an MDP-independent manner [15]. However, it remains obscured whether NOD2 attenuates the HMGB1/TLR4 signaling pathway via the induction of IRF4 in an MDP-independent manner. Additionally, as reported, recruitment of receptor-interacting protein 2 (RIP2, also known as RICK), one of the downstream adaptor kinases, is required for NOD2 dependent inflammatory responses induced by MDP [28, 29]. NOD2 activates RIP2 via CARD-CARD interaction, and RIP2 subsequently polymerizes into filament formation, which is essential for downstream inflammatory responses [30]. Further investigation is warranted to determine whether RIP2 participates in NOD2-dependent inhibition of macrophage activation, and whether there is collaboration between RIP2 and IRF4, considering the latter’s anticipated role as previously reported [31].

As OA involves pathological changes in multiple types of tissues and cells, including macrophages and FLSs in synovial tissue, and chondrocytes in articular cartilage [32], leading to the concept that constituents of joints should be given balanced consideration [18]. In this study, resting macrophages stimulated with HMGB1 were cultured to collect supernatants, which were then added into FLSs and chondrocytes, respectively, to simulate the in vivo intra-articular environment. This approach allowed for the evaluation of the effect of altered NOD2 expression in macrophages on synovial inflammation and cartilage degradation [33]. The inflammatory phenotype of FLSs was characterized by increased invasion and migration, along with polarized formation of lamellipodia with colocalization of phosphorylated focal adhesion kinase (p-FAK) [34, 35]. Chondrocyte inflammatory phenotype involved matrix degradation as well as an imbalance of anabolic and catabolic factors [36, 37]. Given the vital role of macrophages in orchestrating the inflammatory process during OA pathogenesis [38], our findings revealed the significance of NOD2 through in vitro experiments demonstrating that the overexpression of NOD2 alters the paracrine effects of activated macrophages on FLSs and chondrocytes. However, the regulatory mechanism of macrophage NOD2 remains far from comprehensively elucidated.

Recognizing synovial macrophages of joints as potential targets for osteoarthritis prevention and treatment [9, 39], we further performed in vivo experiments to explore feasible interventions in mice. Commonly used OA models in mice include destabilization of medial meniscus (DMM), anterior cruciate ligament transection (ACLT), intra-articular injection of mono-iodoacetate (MIA), and collagenase-induced osteoarthritis (CIOA). CIOA mouse model is characterized by high synovial hyperplasia and, thus, considered more methodologically rigorous in assessing the effect of macrophages [9, 16]. Intra-articular injection of lentiviral vectors was employed as an efficient approach to manipulating the expression of a target gene in vivo [19, 40]. The results suggested that in vivo overexpression of NOD2 via lentiviral vectors significantly mitigates the severity of mouse OA. These findings open possibilities for potential clinical translation in the future, offering avenues for the prevention and treatment of OA.

There are various intra-articular agents for local application to affected joints. Among them, glucocorticoids have been recommended for osteoarthritic patients suffering from pain, due to their potent short-term efficacy in relieving pain [41]. However, they also increase the risk of joint infection and systemic changes after repeated injection. Therefore, intra-articular agents with higher level of safety and efficacy are urgently needed [42]. Gene therapy via lentiviral vectors has emerged as a promising therapeutic option and has been applied in clinical trials since August of 2017. The first lentivirus-mediated cellular therapy, tisagenlecleucel (CTL019, Kymriah), was approved in the USA for the treatment of acute lymphoblastic leukemia in children and adolescents. Theoretically, lentiviral vectors pose risks of insertional mutagenesis, but available clinical data suggest that newer generation vectors strongly reduce the risk, as no relevant case has been reported to date [43]. Thus, lentivirus-mediated macrophage-targeted therapy would represent a promising approach for osteoarthritis.

Modulating the M1/M2 balance by inhibiting M1 macrophages has been developed in preclinical mouse models in osteoarthritis research. Intra-articular injection effectively avoids systemic application, and local application delivers a lower impact on systemic immunity [44]. Additionally, synovial macrophages are derived from circulating macrophages, and there is currently no evidence that synovial macrophage might have the chance to return to circulating blood stream on a clinically significant scale. Although promising, this approach may be associated with pathological consequences, including promoting autoimmune or inflammatory diseases, which has not been definitely established yet [45]. Therefore, a comprehensive understanding of macrophage phenotypic and functional heterogeneity in synovial tissue should be acquired, before we can target specific subsets of macrophages in translational studies to ensure a balance between therapeutic benefits and potential risks in the future.

## Conclusions

In this study, NOD2 emerged as a critical inhibitor of macrophage activation and M1 polarization, particularly in response to HMGB1 stimulation, by acting as a reciprocal modulator of the HMGB1/TLR4 signaling pathway in macrophages. This, in turn, reshapes the paracrine effect of activated macrophages on FLS and chondrocytes during OA pathogenesis. In conclusion, our findings highlighted the impressive potential of NOD2 to be a preventative and therapeutic target in OA, though more in-depth investigations are indispensable to fully elucidate the underlying mechanisms before definitive conclusions can be reached.

### Supplementary Information

**Additional file 1. Supplementary Figure 1. **(A-B) Semi-quantitative analysis of NOD2 and TNF-α at protein level in synovial tissue. (C-G) Semi-quantitative analysis of NF-κB and MAPK pathway activation in macrophages stimulated by HMGB1. ∗ *p* < 0.05, ∗∗ *p* < 0.01 and ∗∗∗ *p* < 0.001. Data were presented as mean ± s.e.m. values. *n* = 3 biologically independent replicates. Student’s *t* test was performed for comparison between two groups, and one-way ANOVA was for multi-group comparison.

**Additional file 2. Supplementary Figure 2. **(A-E) Semi-quantitative analysis of NF-κB and MAPK pathway activation in macrophages treated with TAK-242 and/or si-NOD2. (F-G) MDP enhances HMGB1-induced elevation of TNF-α at mRNA and protein level. (H-L) Semi-quantitative analysis of activation of NF-κB and MAPK pathway dampened by oe-NOD2 in macrophages. ∗ *p* < 0.05, ∗∗ *p* < 0.01 and ∗∗∗ *p* < 0.001. Data were presented as mean ± s.e.m. values. *n* = 3 biologically independent replicates. One-way ANOVA was for multi-group comparison, and two-way ANOVA was for multi-group comparison with additional categorical variables.

**Additional file 3. Supplementary Figure 3. **Semi-quantitative analysis of NF-κB and MAPK pathway activation in FLS dampened by over-expression of macrophage NOD2. Semi-quantitative analysis of anabolic factors including COL2, aggrecan and SOX9, as well as catabolic factors such as ADAMT4/5, MMP3 and MMP13 in chondrocytes. ∗ *p* < 0.05, ∗∗ *p* < 0.01 and ∗∗∗ *p* < 0.001. Data were presented as mean ± s.e.m. values. *n* = 3 biologically independent replicates. Student’s *t* test was performed for comparison between two groups.

**Additional file 4. Supplementary Figure 4. **Transfection of siRNA sequences down-regulates the expression of NOD2 in mRNA level. Lentivirus transfection mediated over-expression of NOD2 in mRNA level. ∗ *p* < 0.05, ∗∗ *p* < 0.01 and ∗∗∗ *p* < 0.001. Data were presented as mean ± s.e.m. values. *n* = 3 biologically independent replicates. Student’s t test was performed for comparison between two groups, and one-way ANOVA was for multi-group comparison.

**Additional file 5: Supplementary Table 1. **Characteristics of participants.

**Additional file 6: Supplementary Table 2. **Primers for real-time PCR.

**Additional file 7: Supplementary Table 3. **List of reagents.

**Additional file 8: Supplementary Table 4.** Sequences of siRNAs.

**Additional file 9: **Gels and blots.

## Data Availability

The data supporting the findings of this study are available from the corresponding author upon reasonable request.

## Abbreviations

OA: Osteoarthritis
FLS: Fibroblast-like synoviocyte
MRI: Magnetic resonance imaging
DAMP: Damage-associated molecular pattern
HMGB1: High-mobility group box-1
PRR: Pattern recognition receptor
TLR: Toll-like receptor
TNF-α: Tumor necrosis factor α
NOD2: Nucleotide-binding oligomerization domain containing 2
CIOA: Collagenase-induced osteoarthritis
SPF: Specific pathogen free
oe-NOD2: NOD2 overexpression
OARSI: Osteoarthritis Research Society International
IHC: Immunohistochemical
PFA: Paraformaldehyde
BSA: Bovine serum albumin
HRP: Horseradish peroxidase
DAB: 3,3′-Diaminobenzidine
DAPI: 4′,6-Diamidino-2-phenylindole
BMDM: Bone marrow derived macrophage
M-CSF: Macrophage colony-stimulating factor
DMEM: Dulbecco’s modified Eagle’s medium
FBS: Fetal bovine serum
MDP: Muramyl dipeptide
PCR: Polymerase chain reaction
RIPA: Radioimmunoprecipitation assay
PMSF: Phenylmethylsulphonyl fluoride
PVDF: Polyvinylidene difluoride
SDS-PAGE: Sodium dodecyl-sulfate polyacrylamide gel electrophoresis
ELISA: Enzyme-linked immunosorbent assay
siRNA: Small interfering RNA
LV: Lentivirus
DEG: Differentially expressed gene
Micro-CT: Micro-computed tomography
ROI: Region of interest
ODS: Optical density score
ANOVA: Analysis of variance
PPIN: Protein-protein interaction network
NLR: NOD-like receptor
NLRC2: NLR with a CARD2
CARD15: Caspase recruitment domain-containing protein 15
LRR: Leucine-rich repeats
p-FAK: Phosphorated focal adhesion kinase
DMM: Destabilization of medial meniscus
ACLT: Anterior cruciate ligament transection
MIA: Mono-iodoacetate
