# Correction: NOD2 attenuates osteoarthritis via reprogramming the activation of synovial macrophages

**DOI:** 10.1186/s13075-024-03261-5

**Published:** 2024-01-13

**Authors:** Changchuan Li, Zhuji Ouyang, Yuhsi Huang, Sipeng Lin, Shixun Li, Jing Xu, Taihe Liu, Jionglin Wu, Peidong Guo, Zhong Chen, Haoyu Wu, Yue Ding

**Affiliations:** Department of Orthopaedic Surgery, Sun Yat-Sen Memorial Hospital, Sun Yat-Sen University, Guangzhou, 510120 China

**Correction: Arthritis Res Ther 25, 249 (2023)**

**https://doi.org/10.1186/s13075-023-03230-4**

Following publication of the original article [1], the authors reported that the following Equal Contribution note was missing in the article “Changchuan Li, Zhuji Ouyang and Yuhsi Huang contributed equally to this paper and should be listed as co-first authors”.

The original article [1] has been updated.
