# Supplementary figures and images for: NOD2 attenuates osteoarthritis via reprogramming the activation of synovial macrophages
Source: Arthritis Res Ther. 2023 Dec 20;25:249. doi: 10.1186/s13075-023-03230-4 (PMC10731717; doi:10.1186/s13075-023-03230-4)

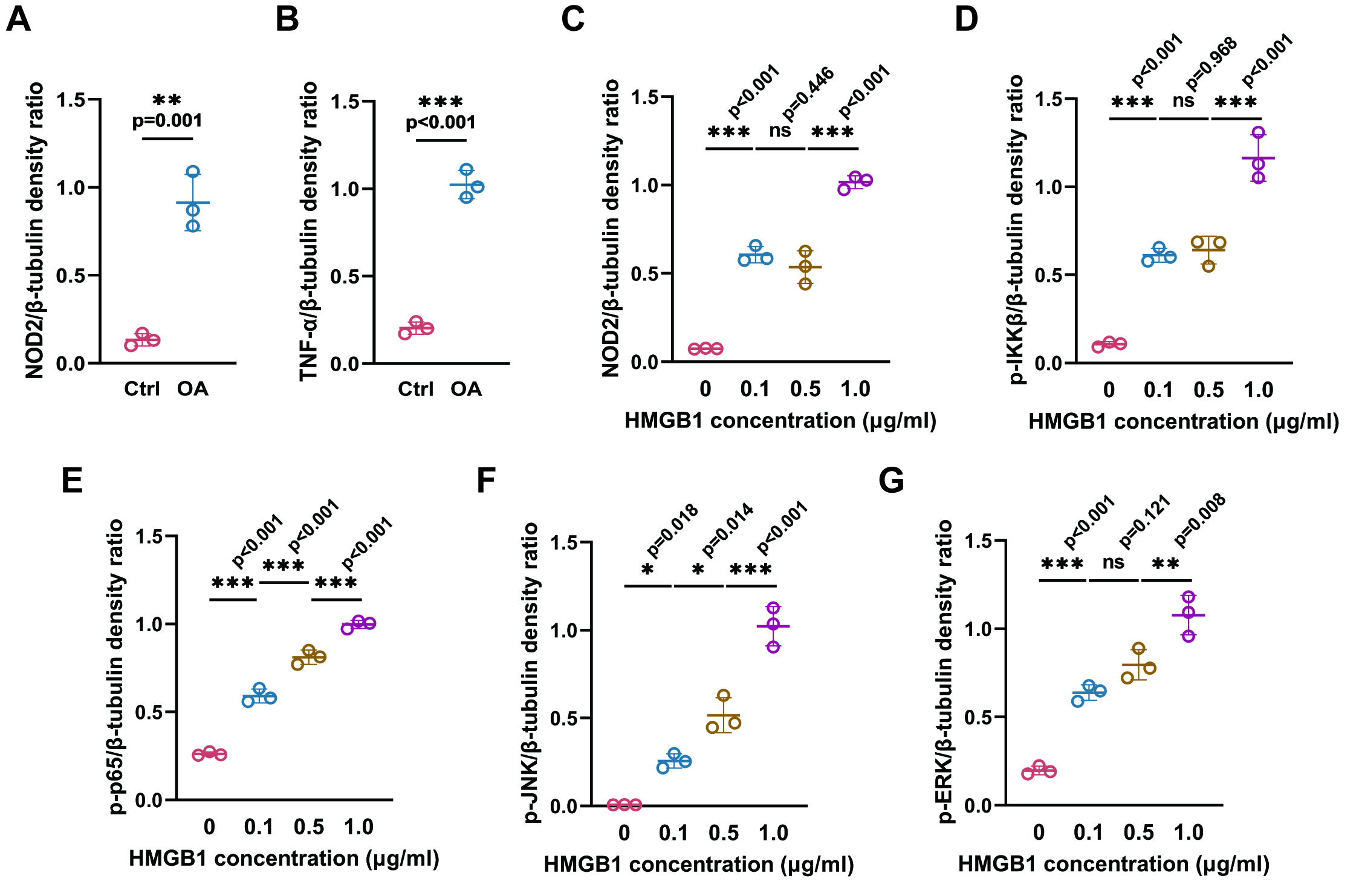

Supplement: Supplementary file 1 — Additional file 1. Supplementary Figure 1. (A-B) Semi-quantitative analysis of NOD2 and TNF-α at protein level in synovial tissue. (C-G) Semi-quantitative analysis of NF-κB and MAPK pathway activation in macrophages stimulated by HMGB1. ∗ p < 0.05, ∗∗ p < 0.01 and ∗∗∗ p < 0.001. Data were presented as mean ± s.e.m. values. n = 3 biologically independent replicates. Student’s t test was performed for comparison between two groups, and one-way ANOVA was for multi-group comparison. [file 13075_2023_3230_MOESM1_ESM.tif]

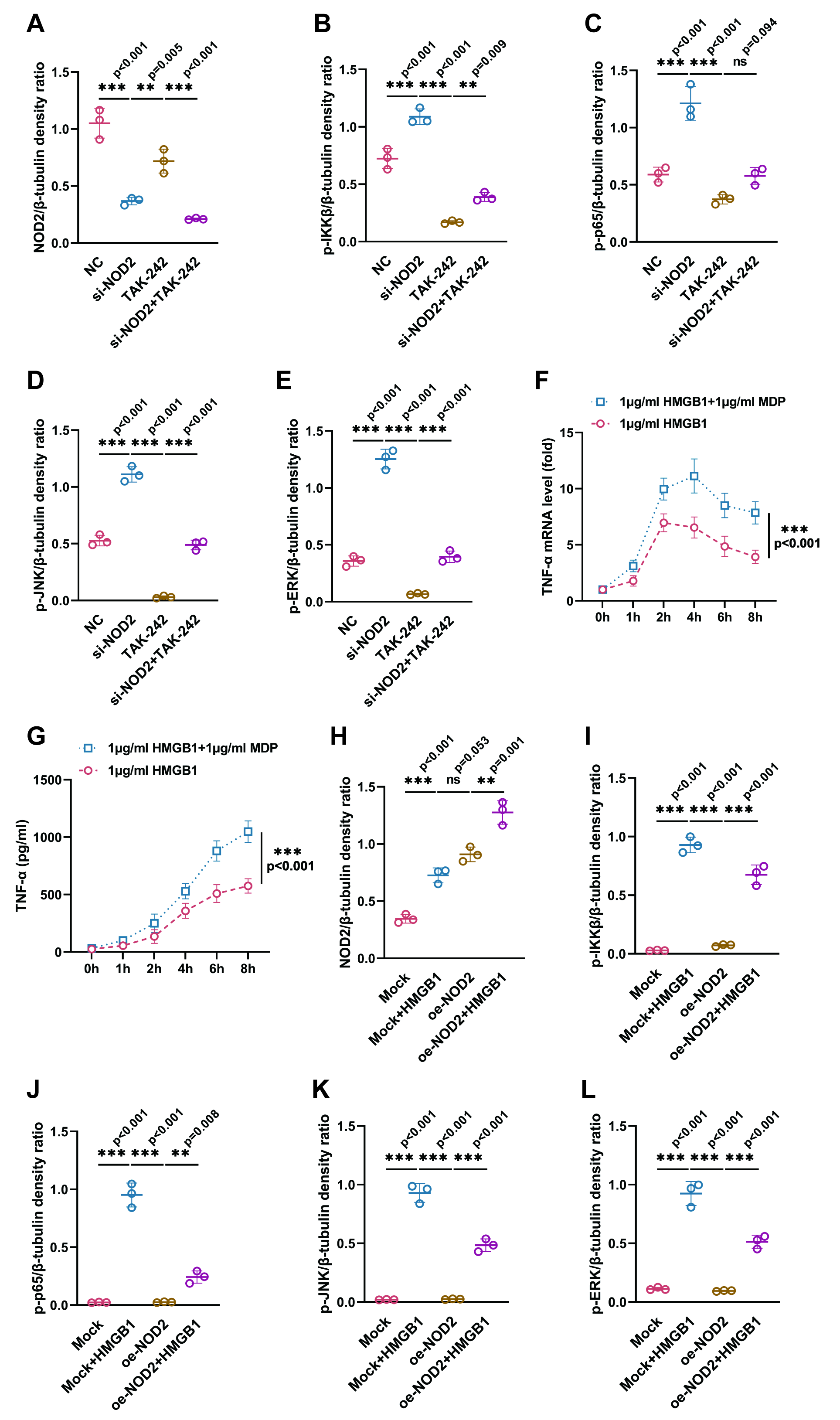

Supplement: Supplementary file 2 — Additional file 2. Supplementary Figure 2. (A-E) Semi-quantitative analysis of NF-κB and MAPK pathway activation in macrophages treated with TAK-242 and/or si-NOD2. (F-G) MDP enhances HMGB1-induced elevation of TNF-α at mRNA and protein level. (H-L) Semi-quantitative analysis of activation of NF-κB and MAPK pathway dampened by oe-NOD2 in macrophages. ∗ p < 0.05, ∗∗ p < 0.01 and ∗∗∗ p < 0.001. Data were presented as mean ± s.e.m. values. n = 3 biologically independent replicates. One-way ANOVA was for multi-group comparison, and two-way ANOVA was for multi-group comparison with additional categorical variables. [file 13075_2023_3230_MOESM2_ESM.tif]

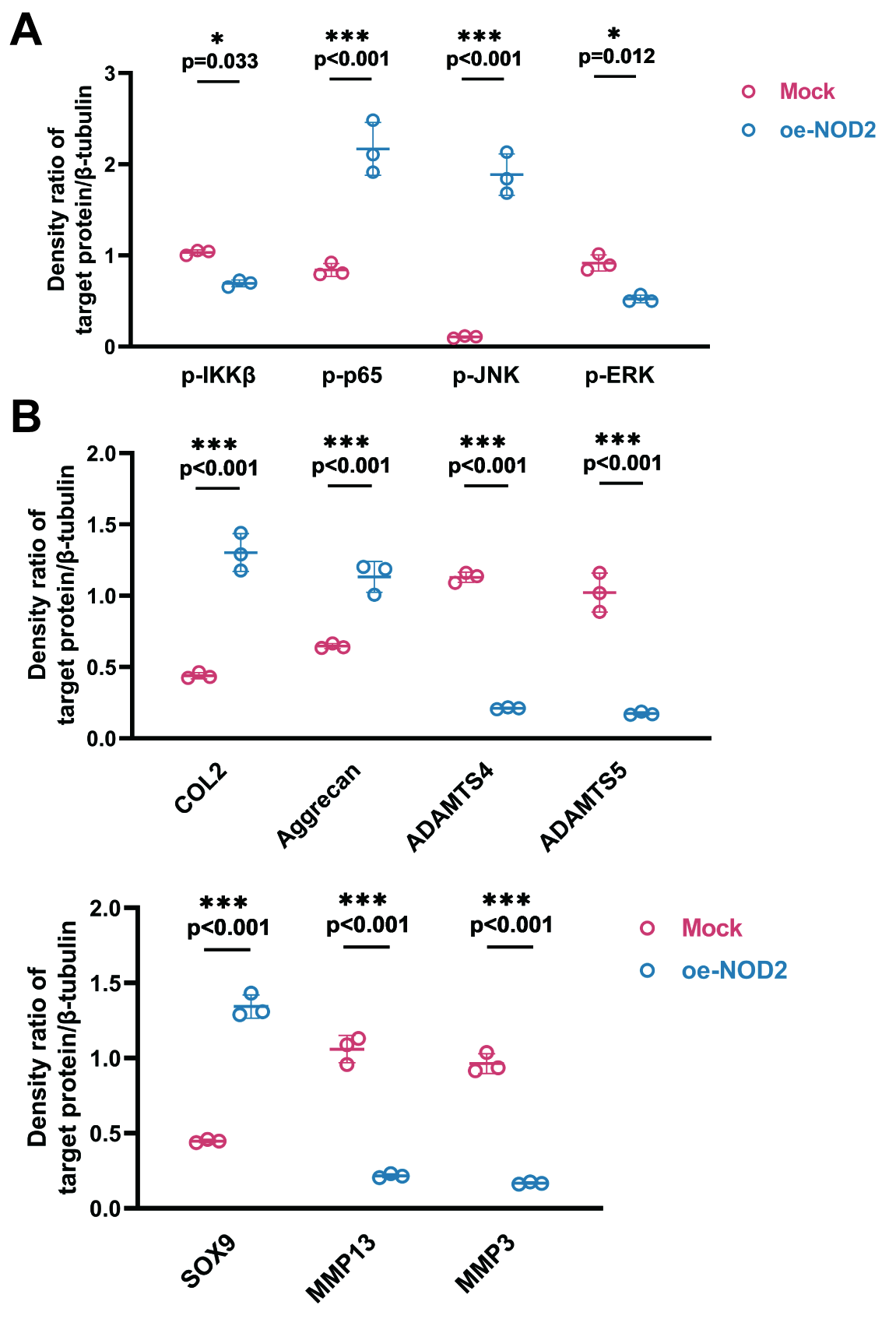

Supplement: Supplementary file 3 — Additional file 3. Supplementary Figure 3. Semi-quantitative analysis of NF-κB and MAPK pathway activation in FLS dampened by over-expression of macrophage NOD2. Semi-quantitative analysis of anabolic factors including COL2, aggrecan and SOX9, as well as catabolic factors such as ADAMT4/5, MMP3 and MMP13 in chondrocytes. ∗ p < 0.05, ∗∗ p < 0.01 and ∗∗∗ p < 0.001. Data were presented as mean ± s.e.m. values. n = 3 biologically independent replicates. Student’s t test was performed for comparison between two groups. [file 13075_2023_3230_MOESM3_ESM.tif]

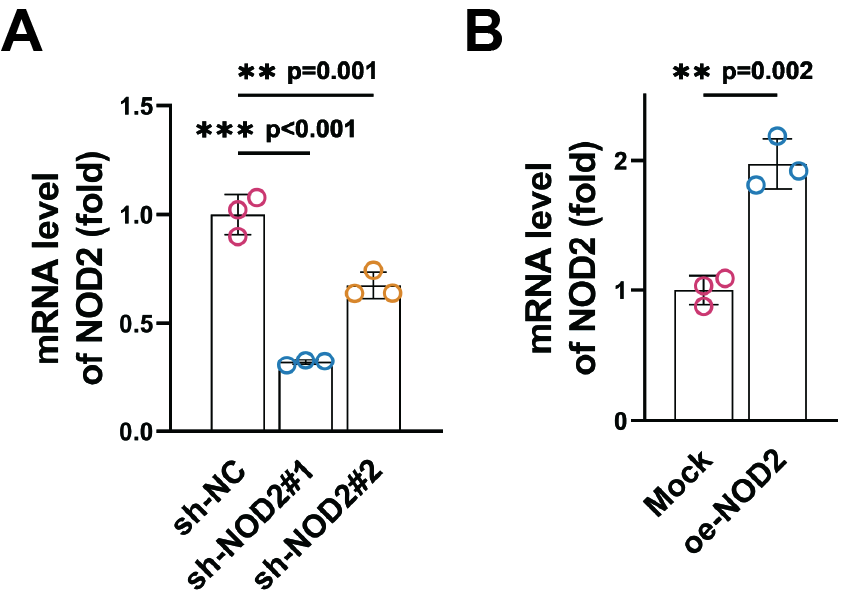

Supplement: Supplementary file 4 — Additional file 4. Supplementary Figure 4. Transfection of siRNA sequences down-regulates the expression of NOD2 in mRNA level. Lentivirus transfection mediated over-expression of NOD2 in mRNA level. ∗ p < 0.05, ∗∗ p < 0.01 and ∗∗∗ p < 0.001. Data were presented as mean ± s.e.m. values. n = 3 biologically independent replicates. Student’s t test was performed for comparison between two groups, and one-way ANOVA was for multi-group comparison. [file 13075_2023_3230_MOESM4_ESM.tif]

Figure 1H

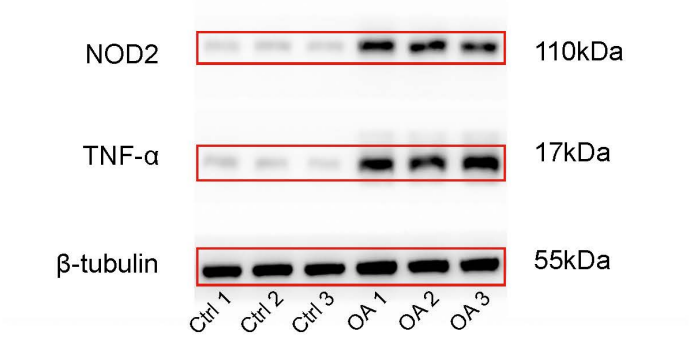

Figure 2H

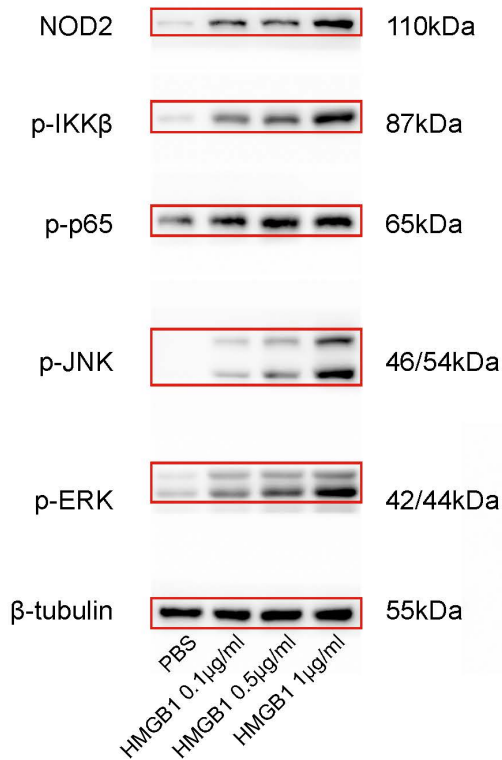

Figure 3D

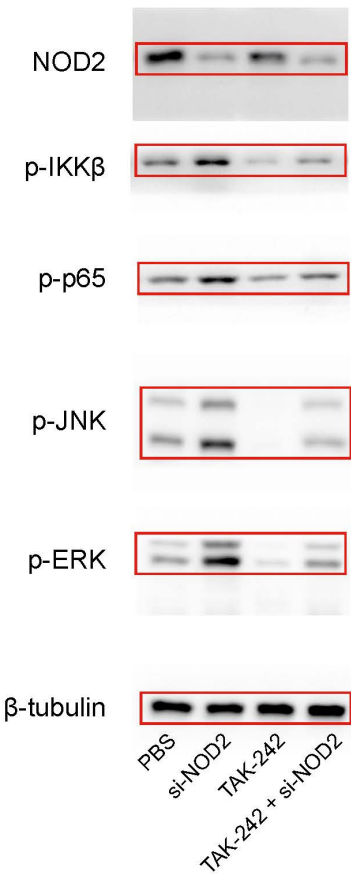

Figure 3I

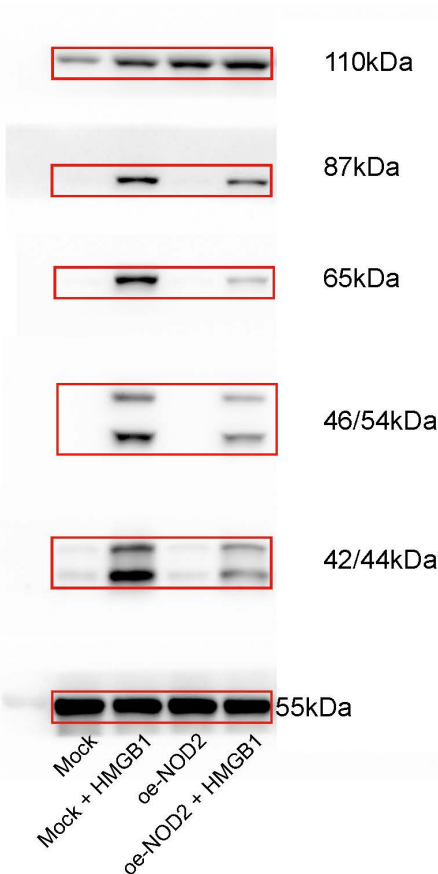

Figure 4B

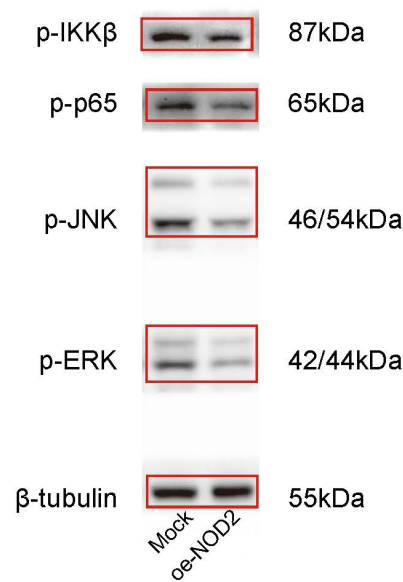

Figure 4I

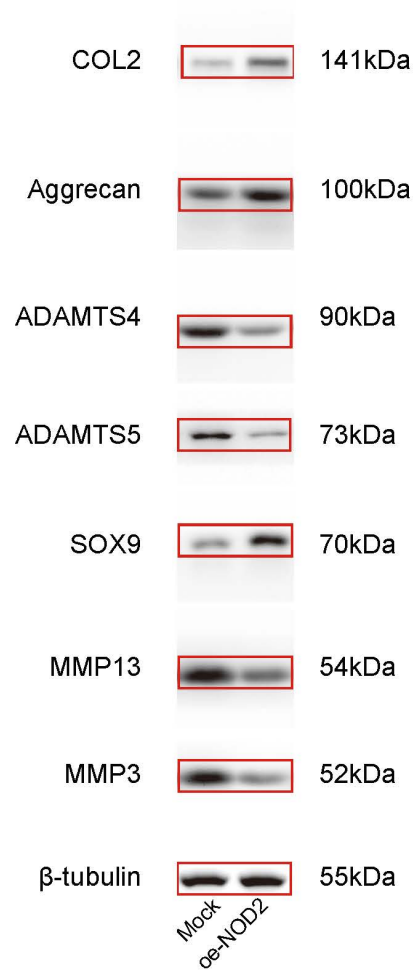

Supplement: Supplementary file 9 — Additional file 9: Gels and blots. [file 13075_2023_3230_MOESM9_ESM.pdf]
