# Supplementary material for: NOD2 attenuates osteoarthritis via reprogramming the activation of synovial macrophages
Source: Arthritis Res Ther. 2023 Dec 20;25:249. doi: 10.1186/s13075-023-03230-4 (PMC10731717; doi:10.1186/s13075-023-03230-4)
Supplement: Supplementary file 5 — Additional file 5: Supplementary Table 1. Characteristics of participants. [file 13075_2023_3230_MOESM5_ESM.docx]

**Supplementary Table 1. Characteristics of participants**

| **Case Number** | **Age** | **Sex** | ^a^ **K-L classification** | **Group** | **Comorbidities** | ^b^ **Krenn Score** |
| --- | --- | --- | --- | --- | --- | --- |
| 1 | 30 | Male | 0 | Ctrl | HBV infection | 1 |
| 2 | 36 | Female | 1 | Ctrl | Thyroid nodule (TIRADS 3) | 0 |
| 3 | 38 | Female | 1 | Ctrl | None | 2 |
| 4 | 67 | Female | 3 | OA | None | 2 |
| 5 | 71 | Male | 2 | OA | HBV infection | 4 |
| 6 | 68 | Female | 3 | OA | Hyperlipidemia | 4 |
| 7 | 58 | Male | 3 | OA | Hypertension | 5 |
| 8 | 69 | Female | 3 | OA | Diabetes mellitus | 5 |
| 9 | 64 | Female | 2 | OA | Hypertension | 3 |
| 10 | 65 | Female | 4 | OA | Osteopenia, hyperuricemia | 6 |
| 11 | 72 | Female | 4 | OA | Atrial fibrillation | 7 |
| 12 | 72 | Male | 4 | OA | Coronary artery disease | 7 |
| 13 | 66 | Male | 4 | OA | Diabetes mellitus | 5 |

^a^ K-L classification: Kellgren-Lawrence classification.

^b^ Interpretation of Krenn score: 0–1, no synovitis; 2–4, low-grade synovitis; and 5–9, high-grade synovitis
