# Supplementary material for: NOD2 attenuates osteoarthritis via reprogramming the activation of synovial macrophages
Source: Arthritis Res Ther. 2023 Dec 20;25:249. doi: 10.1186/s13075-023-03230-4 (PMC10731717; doi:10.1186/s13075-023-03230-4)
Supplement: Supplementary file 6 — Additional file 6: Supplementary Table 2. Primers for real-time PCR. [file 13075_2023_3230_MOESM6_ESM.docx]

**Supplementary Table 2. Primers for real-time PCR**

| **Species** | **Gene** | **ID** |  | **Sequence of primer (5’-3’)** |
| --- | --- | --- | --- | --- |
| Human | NOD2 | 64127 | F | CACCGTCTGGAATAAGGGTACT |
|  |  |  | R | TTCATACTGGCTGACGAAACC |
|  | TNF-α | 7124 | F | GAGGCCAAGCCCTGGTATG |
|  |  |  | R | CGGGCCGATTGATCTCAGC |
| Mouse | NOD2 | 257632 | F | AGCACGTCAGGGAACTACCA |
|  |  |  | R | GGAAGCGAGACTGAGTCAACA |
|  | TNF-α | 21926 | F | CCTGTAGCCCACGTCGTAG |
|  |  |  | R | GGGAGTAGACAAGGTACAACCC |
|  | COL2A1 | 12824 | F | GGGAATGTCCTCTGCGATGAC |
|  |  |  | R | GAAGGGGATCTCGGGGTTG |
|  | Aggrecan | 11595 | F | CCTGCTACTTCATCGACCCC |
|  |  |  | R | AGATGCTGTTGACTCGAACCT |
|  | ADAMTS4 | 240913 | F | ATGGCCTCAATCCATCCCAG |
|  |  |  | R | AAGCAGGGTTGGAATCTTTGC |
|  | ADAMTS5 | 23794 | F | GGAGCGAGGCCATTTACAAC |
|  |  |  | R | CGTAGACAAGGTAGCCCACTTT |
|  | SOX9 | 20682 | F | GAGCCGGATCTGAAGAGGGA |
|  |  |  | R | GCTTGACGTGTGGCTTGTTC |
|  | MMP-3 | 17392 | F | ACATGGAGACTTTGTCCCTTTTG |
|  |  |  | R | TTGGCTGAGTGGTAGAGTCCC |
|  | MMP-13 | 17386 | F | CTTCTTCTTGTTGAGCTGGACTC |
|  |  |  | R | CTGTGGAGGTCACTGTAGACT |
