# Supplementary material for: NOD2 attenuates osteoarthritis via reprogramming the activation of synovial macrophages
Source: Arthritis Res Ther. 2023 Dec 20;25:249. doi: 10.1186/s13075-023-03230-4 (PMC10731717; doi:10.1186/s13075-023-03230-4)
Supplement: Supplementary file 7 — Additional file 7: Supplementary Table 3. List of reagents. [file 13075_2023_3230_MOESM7_ESM.docx]

**Supplementary Table 3. List of reagents.**

| **Reagent** | | **Source** | | | **Catalogue Number** |
| --- | --- | --- | --- | --- | --- |
| **Antibodies** |  | |  |  |  |
| anti-NOD2 | | Proteintech | | | 20980-1-AP |
| anti-TNF-α | | R&D | | | AF-410-NA |
| anti-β-tubulin | | HuaAn Biotechnology | | | ET1602-4 |
| anti-HMGB1 | | ABclonal | | | A19529 |
| anti-p65 | | HuaAn Biotechnology | | | ET1603-12 |
| anti-p-p65 | | ABclonal | | | AP0124 |
| anti-p-IKKβ | | Immunoway | | | YP0637 |
| anti-p-JNK | | Cell Signaling Technology | | | 4668S |
| anti-p-ERK | | Proteintech | | | 28733-1-AP |
| anti-iNOS | | HuaAn Biotechnology | | | ER1706-89 |
| anti-CD206 | | Proteintech | | | 18704-1-AP |
| anti-CD163 | | Abcam | | | ab289979 |
| anti-F4/80 | | HuaAn Biotechnology | | | RT1212 |
| anti-CD80 | | Thermo Fisher Scientific | | | PA5-85913 |
| anti-CD86 | | Thermo Fisher Scientific | | | MA1-10299 |
| anti-Rabbit IgG H&L, Alexa Fluor™ 647 | | Thermo Fisher Scientific | | | A-21245 |
| anti-Rat IgG H&L, Alexa Fluor™ 488 | | Thermo Fisher Scientific | | | A-48262 |
| anti-Rabbit IgG H&L, HRP | | Abcam | | | ab6721 |
| anti-iNOS, PE-Cyanine7 | | Thermo Fisher Scientific | | | 25-5920-82 |
| anti-CD206, APC | | Thermo Fisher Scientific | | | 17-2061-82 |
| anti-p-FAK | | HuaAn Biotechnology | | | ET1610-34 |
| anti-Collagen II | | HuaAn Biotechnology | | | ER1906-49 |
| anti-Aggrecan | | Affinity | | | DF7561 |
| anti-ADAMTS4 | | ABclonal | | | A2525 |
| anti-ADAMTS5 | | ABclonal | | | A2836 |
| anti-SOX9 | | Immunoway | | | YT4371 |
| anti-MMP3 | | Abcam | | | ab151945 |
| anti-MMP13 | | HuaAn Biotechnology | | | ET1702-14 |
| **Chemicals** |  | |  |  |  |
| Collagenase VII | | Sigma-Aldrich | | | C0773 |
| EDTA | | Solarbio | | | E1171 |
| Pepsin | | ZSGB-BIO | | | ZLI-9013 |
| Bovine serum albumin | | Molecular Research Centre, Inc. | | | CCS30014.01 |
| 3,3’-diaminobenzidine (DAB) | | Beyotime Biotechnology | | | P0203 |
| Haematoxylin | | Sigma Aldrich | | | H9627 |
| Neutral balsam | | Solarbio | | | G8590 |
| 4',6-diamidino-2-phenylindole (DAPI) | | HelixGen | | | HNFD-02 |
| Gibco™ Dulbecco’s modified Eagle’s medium (DMEM) | | Thermo Fisher Scientific | | | C11995500BT |
| 10% fetal bovine serum (FBS) | | Thermo Fisher Scientific | | | 10099141 |
| Penicillin & streptomycin | | Thermo Fisher Scientific | | | SV30010 |
| Gibco™ DMEM/F-12 | | Thermo Fisher Scientific | | | 11330032 |
| Recombinant human HMGB1 | | Abcam | | | ab167718 |
| Muramyl dipeptide (MDP) | | InvivoGen | | | 53678-77-6 |
| MG132 | | Cell Signaling Technology | | | 2194S |
| Gibco™ 0.25% Trypsin | | Gibco | | | 15050065 |
| Collagenase II | | Yeasen | | | 40508ES60 |
| RIPA buffer | | Cell Signaling Technology | | | 9806S |
| Phenylmethylsulphonyl fluoride (PMSF) | | Solarbio | | | P0100-1 |
| NuPAGE™ LDS Sample Buffer (4×) | | Thermo Fisher Scientific | | | NP0007 |
| Phosphatase inhibitor cocktail | | CW Biotech | | | CW2383 |
| Protease inhibitor cocktail | | CW Biotech | | | CW2200S |
| Polyvinylidene fluoride (PVDF) membrane | | Millipore | | | IPVH00010 |
| Super ECL Detection Reagent | | Yeasen | | | 36208ES60 |
| Lipofectamine™ RNAiMAX Transfection Reagent | | Thermo Fisher Scientific | | | 13778075 |
| Opti-MEM™ | | Thermo Fisher Scientific | | | 31985088 |
| eBioscience™ IC Fixation Buffer | | Thermo Fisher Scientific | | | 00-8222-49 |
| Perm/Wash Buffer | | BD Biosciences | | | 554723 |
| TRIzol^TM^ Reagent | | Thermo Fisher Scientific | | | 15596026 |
| Safranin O/fast green | | Servicebio | | | G1053 |
| Matrigel^®^ | | Corning, Inc. | | | 356234 |
| Crystal violet solution | | Beyotime | | | C0121 |
| **Kits** |  | |  |  |  |
| Mouse TNF-α ELISA kit | | Neobioscience | | | EMC102a |
| RNAiso Plus reagent kit | | TaKaRa Bio | | | 9109 |
| PrimeScript™ RT Master Mix reagent kit | | TaKaRa Bio | | | RR036D |
| UNICON^TM^ qPCR SYBR^®^ Green Master Mix | | Yeasen | | | 11198ES08 |
| Pierce™ BCA Protein Assay Kit | | Thermo Fisher Scientific | | | 23225 |
