# Supplementary material for: NOD2 attenuates osteoarthritis via reprogramming the activation of synovial macrophages
Source: Arthritis Res Ther. 2023 Dec 20;25:249. doi: 10.1186/s13075-023-03230-4 (PMC10731717; doi:10.1186/s13075-023-03230-4)
Supplement: Supplementary file 8 — Additional file 8: Supplementary Table 4. Sequences of siRNAs. [file 13075_2023_3230_MOESM8_ESM.docx]

**Supplementary Table 4. Sequences of siRNAs**

| **Gene** | **Sequences (5’-3’)** |
| --- | --- |
| NOD2-shRNA#1 | CTCGCTTCCTCAGTACTTACTCGAGTAAGTACTGAGGAAGCGAG |
| NOD2-shRNA#2 | GCACAGAGTTGCAACTGAATTCTCGAGTTCAGTTGCAACTCTGTGC |
| NOD2-siRNA#1 | CTCGCTTCCTCAGTACTTATT |
| NOD2-siRNA#2 | GCACAGAGTTGCAACTGAATT |
